# Cannabis constituents interact at the drug efflux pump BCRP to markedly increase plasma cannabidiolic acid concentrations

**DOI:** 10.1038/s41598-021-94212-6

**Published:** 2021-07-22

**Authors:** Lyndsey L. Anderson, Maia G. Etchart, Dilara Bahceci, Taliesin A. Golembiewski, Jonathon C. Arnold

**Affiliations:** 1 Brain and Mind Centre, Discipline of Pharmacology, Faculty of Medicine and Health, The University of Sydney, Sydney, Australia; 2 Lambert Initiative for Cannabinoid Therapeutics, Brain and Mind Centre, The University of Sydney, 94 Mallett St, Camperdown, NSW 2050 Australia

**Keywords:** Pharmacokinetics, Pharmacology

## Abstract

Cannabis is a complex mixture of hundreds of bioactive molecules. This provides the potential for pharmacological interactions between cannabis constituents, a phenomenon referred to as “the entourage effect” by the medicinal cannabis community. We hypothesize that pharmacokinetic interactions between cannabis constituents could substantially alter systemic cannabinoid concentrations. To address this hypothesis we compared pharmacokinetic parameters of cannabinoids administered orally in a cannabis extract to those administered as individual cannabinoids at equivalent doses in mice. Astonishingly, plasma cannabidiolic acid (CBDA) concentrations were 14-times higher following administration in the cannabis extract than when administered as a single molecule. In vitro transwell assays identified CBDA as a substrate of the drug efflux transporter breast cancer resistance protein (BCRP), and that cannabigerol and Δ^9^-tetrahydrocannabinol inhibited the BCRP-mediated transport of CBDA. Such a cannabinoid-cannabinoid interaction at BCRP transporters located in the intestine would inhibit efflux of CBDA, thus resulting in increased plasma concentrations. Our results suggest that cannabis extracts provide a natural vehicle to substantially enhance plasma CBDA concentrations. Moreover, CBDA might have a more significant contribution to the pharmacological effects of orally administered cannabis extracts than previously thought.

## Introduction

Cannabis is a complex mixture of bioactive molecules including cannabinoids, terpenoids and flavonoids. There are around > 120 terpenophenolic cannabinoids found in cannabis including the major cannabinoids, Δ^9^-tetrahydrocannabinol (Δ^9^-THC) and cannabidiol (CBD). CBD and Δ^9^-THC have undergone extensive pharmacological characterization; however, other cannabinoids also possess biological activity and may contribute to the pharmacological effects of medicinal cannabis^1, 2^. Indeed, there is emerging evidence that acidic precursors of the neutral forms of the cannabinoids have pharmacological activity^3–5^. These acidic precursors are abundant in the plant and can achieve appreciable plasma concentrations following the ingestion of medicinal cannabis products^4, 5^. Medicinal cannabis products contain a multitude of both acidic and neutral cannabinoids amongst other phytochemicals, each with a complex pharmacology, so there is the potential for interactions between cannabis constituents.

A prevailing sentiment in the cannabis community of entrepreneurs and patient advocates is the notion of the "entourage effect," which postulates that effects of the whole cannabis plant are greater than the sum of its individual parts due to an interaction between its phytochemical constituents. While many favour the view that cannabis constituent interactions engender greater beneficial effects, such interactions might also enhance deleterious effects. Few studies have addressed the "entourage effect" hypothesis; however, there is growing evidence that the effects of full-spectrum cannabis extracts may not be attributed to an individual constituent. Recent studies have shown greater antitumor effects with Δ^9^-THC-rich cannabis extracts compared to purified Δ^9^-THC^6, 7^. Several preclinical studies have examined the effects of cannabinoids administered in combination. CBD enhanced the anticonvulsant action of Δ^9^-THC against thermally-induced seizures in a mouse model of Dravet syndrome, although the combination of CBD and Δ^9^-THC had proconvulsant effects on spontaneous seizures^8^. High potency synergy was reported for CBD and Δ^9^-THC against allodynia in a mouse model of neuropathic pain with less motor side-effects^9^.

Cannabis-based products have gained widespread media attention over the last decade due to artisanal CBD-dominant extracts being reported to have remarkable anticonvulsant effects in children with intractable epilepsies^10^. More recently there has been a "CBD craze", with a substantial increase in demand for cannabis-based products which are perceived to treat a myriad of health conditions^11^. These products, which contain a multitude of cannabinoids, are administered at much lower doses than purified forms of CBD and Δ^9^-THC that have been shown to be effective in clinical trials^10, 11^. Consistent with the “entourage effect” hypothesis it has been suggested that pharmacodynamic interactions between phytochemicals in cannabis occur due to a concerted action at an individual drug target or via activating complementary pathways. However, an "entourage effect" could also arise from pharmacokinetic interactions between components in medicinal cannabis, whereby the absorption, distribution, metabolism and excretion of the cannabinoids are affected. Indeed, pharmacokinetic interactions have been observed between cannabinoids with co-administration resulting in increased cannabinoid concentrations in tissues and blood^8, 12, 13^.

In the present study we aimed to explore the potential for pharmacokinetic interactions between cannabinoids within a full-spectrum cannabis extract administered orally. Oral administration is an increasingly preferred mode of delivery of cannabis oils and is the dominant mode of delivery for childhood epilepsy patients^10, 11, 14^. We compared the pharmacokinetic parameters of cannabinoids administered as an extract to those when administered as an individual compound at equivalent doses.

## Results

### The pharmacokinetic profiles of various cannabinoids administered in a full-spectrum cannabis extract differ substantially from cannabinoids administered as single molecules at equivalent doses

**Figure 1:**
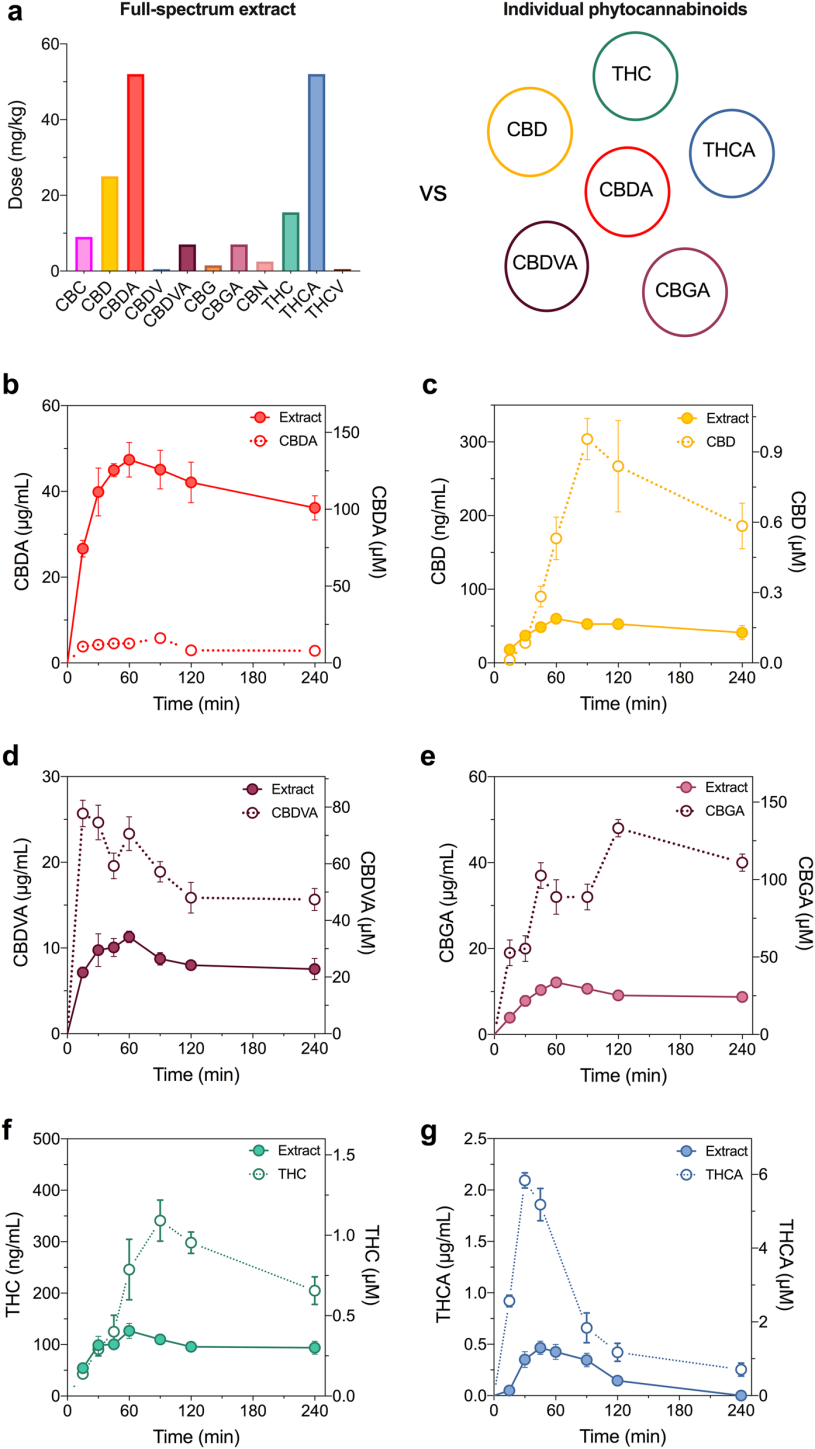
Pharmacokinetic analysis of orally administered cannabinoids in mouse plasma. Cannabinoids were administered orally as either a full-spectrum cannabis extract or individually at equivalent doses to those in the full-spectrum extract. (**a**) Dose and profile of cannabinoids within the full-spectrum extract (left panel) vs. the cannabinoids administered individually (right panel). Concentration–time curves for (**b**) CBDA, (**c**) CBD (**d**) CBDVA (**e**) CBGA (**f**) Δ^9^-THC and (**f**) Δ^9^-THCA. Concentrations are depicted as both mass concentrations (left y-axis) and molar concentrations (right y-axis) for each cannabinoid administered as a full-spectrum extract (solid symbols) or as an individual compound (open symbols). Data are expressed as means ± SEM, with n = 4–5 per time point.

The cannabinoid profile of the full-spectrum cannabis extract was diverse, containing the greatest quantities of cannabidiolic acid (CBDA), Δ^9^-tetrahydrocannabinolic acid (Δ^9^-THCA), CBD and Δ^9^-THC (Fig. 1a). To infer whether compound-compound interactions alter the pharmacokinetic profile of the cannabinoids in the full-spectrum extract, we compared the profiles of the cannabinoids administered in a full-spectrum extract to those of the cannabinoids administered as individual components (Fig. 1). The full-spectrum extract was administered orally as a single dose and plasma cannabinoid concentrations were quantified. CBC, cannabidivarin (CBDV), cannabigerol (CBG), cannabinol (CBN) and Δ^9^-tetrahydrocannabivarin (Δ^9^-THCV) were not detected in plasma following oral administration of the full-spectrum extract so no further pharmacokinetic characterization of these cannabinoids was conducted.

Six cannabinoids were detected in plasma following oral administration of the full-spectrum extract: CBD, CBDA, cannabidivarinic acid (CBDVA), cannabigerolic acid (CBGA), Δ^9^-THC and Δ^9^-THCA. Each was then administered orally as an individual compound at an equivalent dose to that found in the full-spectrum extract.

Astonishingly, the plasma CBDA concentrations that were observed following administration of the full-spectrum cannabis extract were substantially *higher* than that observed when CBDA was administered as a single molecule at an equivalent dose (Fig. 1b). Accordingly, the plasma C_max_ value of CBDA within the full-spectrum extract (47 ± 4 µg/mL) was substantially higher than the C_max_ value achieved as a single molecule (6 ± 1 µg/mL). Moreover, total exposure of CBDA as determined by AUC values when administered in a full-spectrum extract was nearly 14 × the exposure that was observed following its administration as an individual compound (Fig. 1b, Table 1). Conversely, the plasma concentrations of CBD, CBDVA, CBGA, Δ^9^-THC and Δ^9^-THCA following administration of the full-spectrum extract were substantially *lower* when they were administered as single molecules at equivalent doses; the total plasma exposure of each was nearly 2–4 ×  lower following administration in the full-spectrum extract to when administered individually (Fig. 1c–g, Table 1).

**Table 1:** Pharmacokinetics of cannabinoids in plasma following oral administration as a full-spectrum extract or individual cannabinoid.

	CBD, 25 mg/kg		CBDA, 50 mg/kg		CBDVA, 7 mg/kg		CBGA, 7 mg/kg		THC, 15 mg/kg		THCA, 50 mg/kg	
	Extract	CBD	Extract	CBDA	Extract	CBDVA	Extract	CBGA	Extract	THC	Extract	THCA
C_max_ (ng/mL)	60 ± 6	304 ± 28	47 ± 4 µg/mL	6 ± 1 µg/mL	11 ± 1 µg/mL	26 ± 2 µg/mL	12 ± 1 µg/mL	48 ± 2 µg/mL	127 ± 15	341 ± 40	465 ± 64	2094 ± 74
t_max_ (min)	60	90	60	90	60	15	60	120	60	90	45	30
t_1/2_ (min)	484	217	310	198	120	150	298	n.d	330	210	46	37
AUC (µg min/mL)	43	104	22,779	1635	3844	7459	5172	8667*	62	116	44	173

*n.d.* not determined.

*AUC does not include terminal phase.

Absorption of the cannabinoids into plasma following oral administration of the full-spectrum extract was slow with t_max_ values of 45–60 min (Table 1). While CBD, CBDA, CBGA and Δ^9^-THC were all maximally absorbed (t_max_) by 60 min when administered as a full-spectrum extract, plasma t_max_ values were delayed (90–120 min) when each were administered individually (Fig. 1, Table 1). In contrast, absorption of CBDVA (t_max_ 15 min) and Δ^9^-THCA (t_max_ 30 min) was more rapid as individual cannabinoids compared to within the full-spectrum extract (t_max_ values 60 and 45 min, respectively).

Interestingly, when administered in the full-spectrum extract the cannabinoids had relatively long half-lives, 484 min (CBD), 310 min (CBDA), 120 min (CBDVA), 298 min (CBGA) and 330 min (Δ^9^-THC), with the exception of Δ^9^-THCA (t_1/2_ 46 min) and were, for the most part, longer than those when the cannabinoids were administered individually, 217 min (CBD), 198 min (CBDA), 210 min (Δ^9^-THC) and 37 min (Δ^9^-THCA). The half-life of CBDVA, however, was slightly longer when administered as a single compound (150 min vs. 120 min). Since the t_max_ of CBGA was 120 min, there were not enough data points in the elimination phase to calculate a t_1/2_ value.

Overall, the differing pharmacokinetic parameters for the cannabinoids when administered in a full-spectrum extract compared to as individual compounds indicate pharmacokinetic interactions might be occurring between cannabinoids within the full-spectrum extract.

### CBDA, CBD, CBDVA, CBG and Δ^9^-THC are BCRP substrates

Drug transporters, including ATP-binding cassette (ABC) transporters, facilitate the movement of substrates across biological membranes and transporter-mediated interactions within the intestinal lumen can profoundly affect oral bioavailability of co-administered drugs. The best characterised ABC transporters, P-glycoprotein and breast cancer resistance protein (BCRP), are located on the apical surface of epithelial cells in the intestine and extrude substrates back into the intestinal lumen, thereby limiting systemic absorption. Cannabinoids are both substrates and/or inhibitors of ABC transporters so we aimed to examine whether the converging action of the cannabinoids on these transporters might provide a mechanism for the pharmacokinetic interaction observed here^15–19^.

We first aimed to determine whether the cannabinoids found in the full-spectrum cannabis extract were substrates of P-glycoprotein and BCRP by using MDCK cells expressing human P-glycoprotein or BCRP in vitro*.* Transwell assays were conducted to assess bidirectional transport of the cannabinoids detected in plasma following administration of the full-spectrum cannabis extract (CBD, CBDA, CBDVA, CBGA, Δ^9^-THC and Δ^9^-THCA), including the respective neutral compounds (CBDV and CBG) across wildtype, BCRP and P-glycoprotein MDCK cell monolayers. Permeability in the basolateral to apical (B > A) and apical to basolateral (A > B) directions were determined for each of the transporters and compared to respective permeabilities in the wildtype control cells. BCRP and P-glycoprotein preferentially transport substrates in the B > A direction.

**Figure 2:**
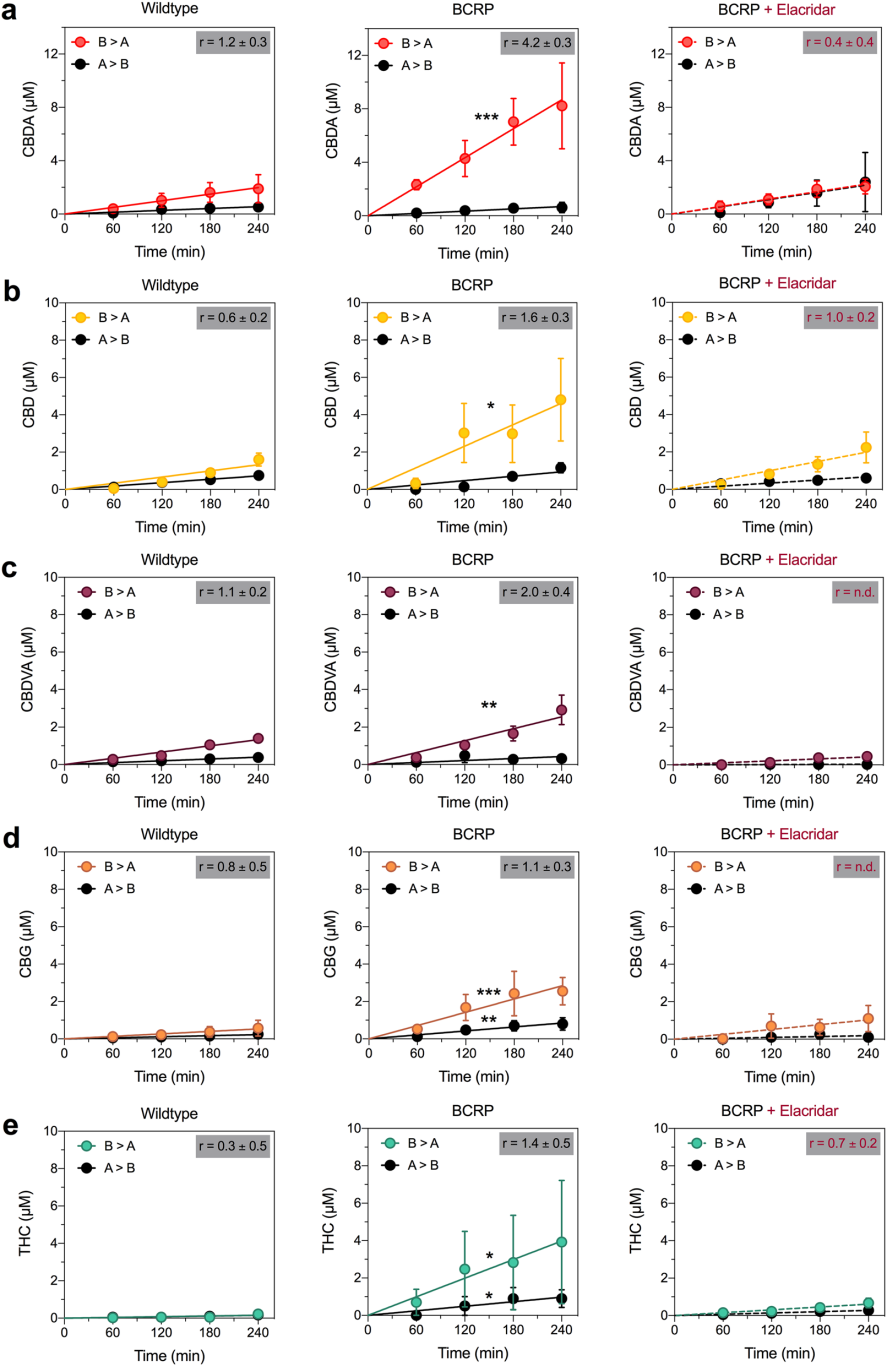
CBD, CBDA, CBDVA, CBG and Δ^9^-THC are substrates of BCRP. Concentration–time curves for (**a**) CBDA, (**b**) CBD, (**c**) CBDVA, (**d**) CBG and (**e**) Δ^9^-THC in wildtype (left panel) and BCRP-expressing (middle panel) MDCK cells in the basolateral to apical (B > A) and apical to basolateral (A > B) directions. Right panels represent concentration–time curves for cannabinoids in cells expressing BCRP in the presence of the inhibitor elacridar (dashed lines). Data are expressed as means ± SEM, with n = 4 per time point. Curves represent fits to a linear regression and transport efflux ratios (r) are listed (*p < 0.05, **p < 0.005, ***p < 0.0005 compared to wildtype; Extra sum-of-squares F test).

CBD, CBDA and CBDVA were BCRP substrates, as the cell permeabilities of these compounds in the B > A direction was significantly greater in the BCRP overexpressing cells than in wildtype cells (CBD, *p* = 0.0105; CBDA, *p* = 0.0002; CBDVA, *p* = 0.0028) without impacting the A > B direction (Fig. 2a–c, Table 2). Moreover, the efflux ratios (r) calculated for each cannabinoid further support the characterization of CBD, CBDA and CBDVA as BCRP substrates, as they exceeded the generally accepted transport ratio threshold of 1.5 for BCRP substrates^20, 21^. Additionally, the BCRP inhibitor elacridar (10 µM) significantly inhibited the transport of these three cannabinoid substrates (CBD, *p* = 0.0249; CBDA, *p* < 0.0001; CBDVA, *p* < 0.0001; Fig. 2a-c).

**Table 2:** Permeabilities of cannabinoids in wildtype, BCRP and P-glycoprotein MDCK cells.

Wildtype				BCRP			P-glycoprotein		
Substrate	P (B > A)	P (A > B)	r	P (B > A)	P (A > B)	r	P (B > A)	P (A > B)	r
CBD	25 ± 3	41 ± 5	0.6 ± 0.2	86 ± 20*	52 ± 8	1.6 ± 0.3	15 ± 3	25 ± 7	0.6 ± 0.3
CBD + Elacridar	–	–	–	37 ± 6^a^	37 ± 6	1.0 ± 0.2	–	–	–
CBDA	37 ± 9	31 ± 8	1.2 ± 0.3	162 ± 24***	38 ± 11	4.2 ± 0.3	38 ± 4	24 ± 7	1.5 ± 0.3
CBDA + Elacridar	–	–	–	42 ± 6^b^	121 ± 48	0.3 ± 0.3	–	–	–
CBDV	44 ± 5	43 ± 5	1.0 ± 0.2	90 ± 9***	63 ± 14	1.4 ± 0.2	34 ± 3	30 ± 8	1.1 ± 0.3
CBDV + Elacridar	–	–	–	68 ± 7*	49 ± 8	1.4 ± 0.2	–	–	–
CBDVA	25 ± 2	22 ± 3	1.1 ± 0.2	47 ± 6**	24 ± 9	2.0 ± 0.4	22 ± 2	14 ± 4	1.6 ± 0.3
CBDVA + Elacridar	n/a	n/a	n/a	8 ± 1^b^	n.d	n.d	–	–	–
CBG	10 ± 3	12 ± 5	0.8 ± 0.5	53 ± 10***	47 ± 9**	1.1 ± 0.3	14 ± 2	19 ± 5	0.7 ± 0.3
CBG + Elacridar	–	–	–	19 ± 6^a^	n.d	n.d	–	–	–
CBGA	1 ± 1	n.d	n.d	5 ± 1	n.d	n.d	3 ± 1	5 ± 2	0.7 ± 0.5
Δ^9^-THC	3 ± 1	9 ± 3	0.3 ± 0.3	74 ± 28*	54 ± 17*	1.4 ± 0.5	4 ± 1	9 ± 3	0.5 ± 0.4
Δ^9^-THC + Elacridar	–	–	–	11 ± 2**^,a^	15 ± 3^a^	0.7 ± 0.2	–	–	–
Δ^9^-THCA	n.d	n.d	n.d	6 ± 2	n.d	n.d	8 ± 3	17 ± 5	0.5 ± 0.4

*P* Permeability calculations (× 10^−5^ cm/s).

*n.d.* not determined; slope of concentration–time curve was not significantly different from zero.

*p < 0.05, **p < 0.005, ***p < 0.0005 compared to corresponding wildtype condition.

^a^*p* < 0.05, ^b^*p* < 0.0001 compared to without inhibitor.

CBG and Δ^9^-THC were also weak substrates of BCRP (Fig. 2d,e Table 2). BCRP permeabilities in the B > A direction were significantly greater than those of wildtype cells (CBG, *p* = 0.0002 and Δ^9^-THC, *p* = 0.0183; Fig. 2, Table 2). Because permeabilities in the A > B direction for cells expressing BCRP were also significantly greater than for wildtype cells (CBG, *p* = 0.0018 and Δ^9^-THC, *p* = 0.0125) the transport ratios for CBG (1.1 ± 0.3) and Δ^9^-THC (1.4 ± 0.5) were below the threshold for BCRP substrates. However, because BCRP-mediated transport of both CBG and Δ^9^-THC was significantly inhibited by elacridar (CBG, *p* = 0.0118 and Δ^9^-THC, *p* = 0.0361) these cannabinoids were deemed weak BCRP substrates (Fig. 2d,e). The B > A directional permeability of CBDV in cells expressing BCRP was significantly greater that that of wildtype cells (*p* = 0.0001) suggesting CBDV might be a BCRP substrate; however, CBDV transport was not inhibited by elacridar so CBDV was not considered a substrate of BCRP (Table 2).

CBD, CBDA, CBDVA, CBG and Δ^9^-THC were not P-glycoprotein substrates, with transport ratios < 2.5, which is the accepted threshold for P-glycoprotein substrates (Table 2)^20^. CBGA, CBDV and Δ^9^-THCA were not substrates of either BCRP or P-glycoprotein. Transport ratios for CBGA and Δ^9^-THCA could not be calculated in wildtype or BCRP-expressing cells since rates of transport were not significantly different from zero. While transport of CBGA and Δ^9^-THCA was achieved in cells expressing P-glycoprotein, it was minimal and transport ratios were 0.7 ± 0.5 and 0.5 ± 0.4, respectively (Table 2).

### CBG and Δ^9^-THC inhibit BCRP-mediated transport of CBDA

**Figure 3:**
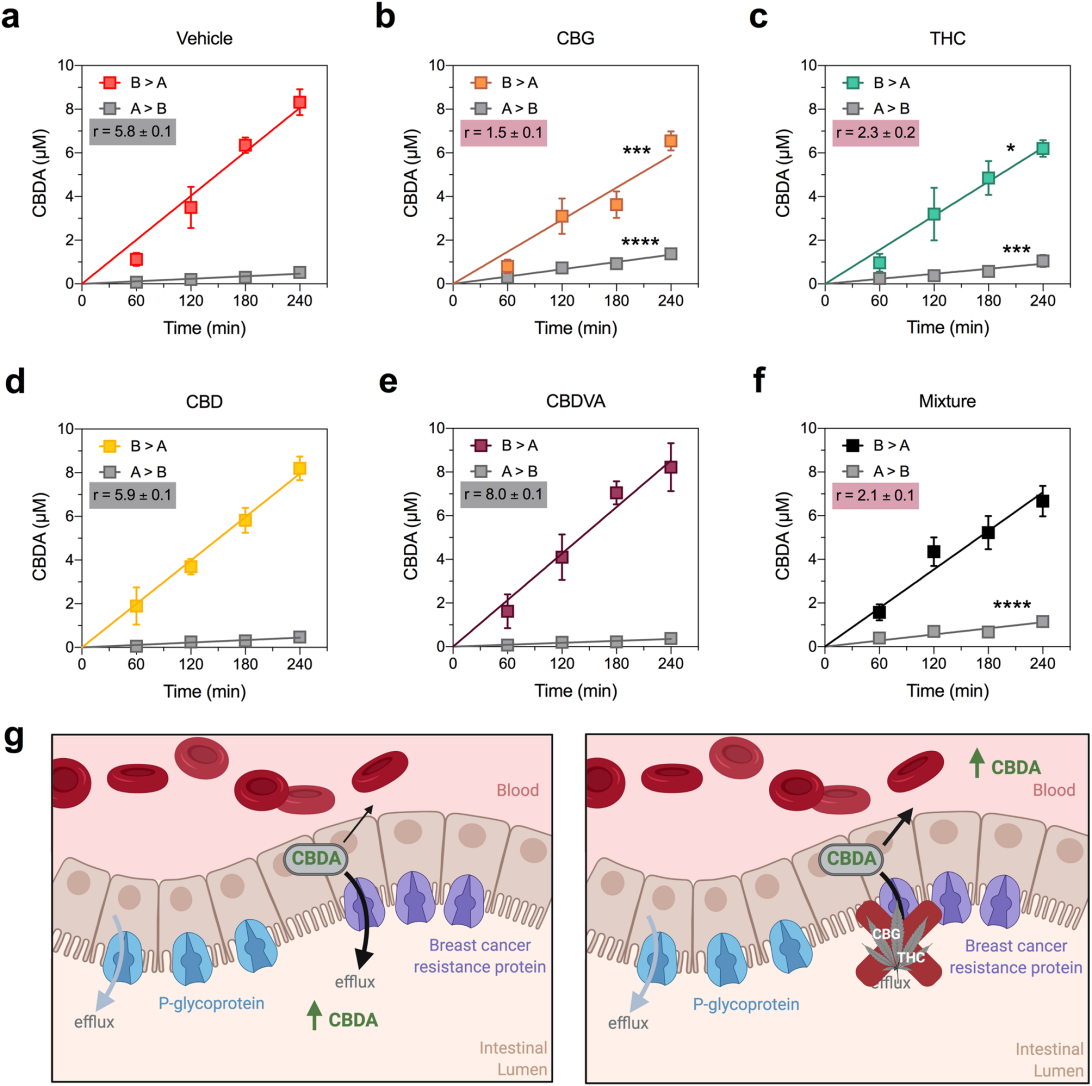
CBG and Δ^9^-THC inhibit BCRP-mediated transport of CBDA. Concentration–time curves for CBDA in the presence of (**a**) vehicle, (**b**) CBG, (**c**) Δ^9^-THC and (**d**) CBD, (**e**) CBDVA and (**f**) a mixture of all four cannabinoids in the basolateral to apical (B > A) and apical to basolateral (A > B) directions in cells expressing BCRP. Cannabinoids were tested at 10 µM. CBG and Δ^9^-THC significantly inhibit (red shading) transport of CBDA. Data are expressed as means ± SEM, with n = 4 per time point. Curves represent fits to a linear regression and transport efflux ratios (r) are listed (*p < 0.05, ***p < 0.0005, ****p < 0.0001 compared to vehicle; Extra sum-of-squares F test). (**g**) Schematic of CBDA efflux by BCRP located in the intestinal lumen when administered alone (left panel) or as a full-spectrum cannabis extract where its efflux is inhibited by CBG and Δ^9^-THC (right panel). Schematic created using BioRender.com.

Since CBDA was identified as a BCRP substrate, it is possible that cannabinoids within the full-spectrum extract inhibited BCRP-mediated efflux of CBDA in the intestinal lumen, which would enhance plasma CBDA exposure following oral dosing with the full-spectrum extract. Hence, we investigated whether the cannabinoids identified as BCRP substrates (CBD, CBDVA, CBG and Δ^9^-THC) inhibited BCRP-mediated transport of CBDA, as substrates may competitively inhibit the transport of other substrates. Rates of CBDA transport in both the B > A and A > B directions were significantly inhibited by 10 µM CBG and Δ^9^-THC (B > A: *p* = 0.0015 and *p* = 0.0131, respectively; A > B: *p* < 0.0001 and *p* = 0.0007, respectively) resulting in lower transport ratios (Fig. 3, Table 3). Neither CBD nor CBDVA affected CBDA transport via BCRP. CBDA permeability was also examined in the presence of a mixture of all four cannabinoids and a lower transport ratio was observed; however, the mixture only significantly increased A > B permeability (*p* < 0.0001) (Fig. 3f, Table 3).

**Table 3:** BCRP permeabilities of CBDA in the presence of cannabinoids.

	CBDA		
Inhibitor	P (B > A)	P (A > B)	r
Vehicle	60 ± 3	10 ± 1	5.8 ± 0.1
CBD	59 ± 3	10 ± 1	5.9 ± 0.1
CBDVA	63 ± 4	8 ± 1	8.0 ± 0.1
CBG	44 ± 3***	30 ± 2****	1.5 ± 0.1
THC	47 ± 4*	21 ± 3***	2.3 ± 0.2
Mixture	53 ± 4	25 ± 3****	5.8 ± 0.1

*P* Permeability calculations (× 10^−5^ cm/s).

*p < 0.05, ***p < 0.0005, ****p < 0.0001 compared to vehicle.

### CBD but not the other phytocannabinoids modestly inhibited BCRP-mediated transport of prazosin

**Figure 4:**
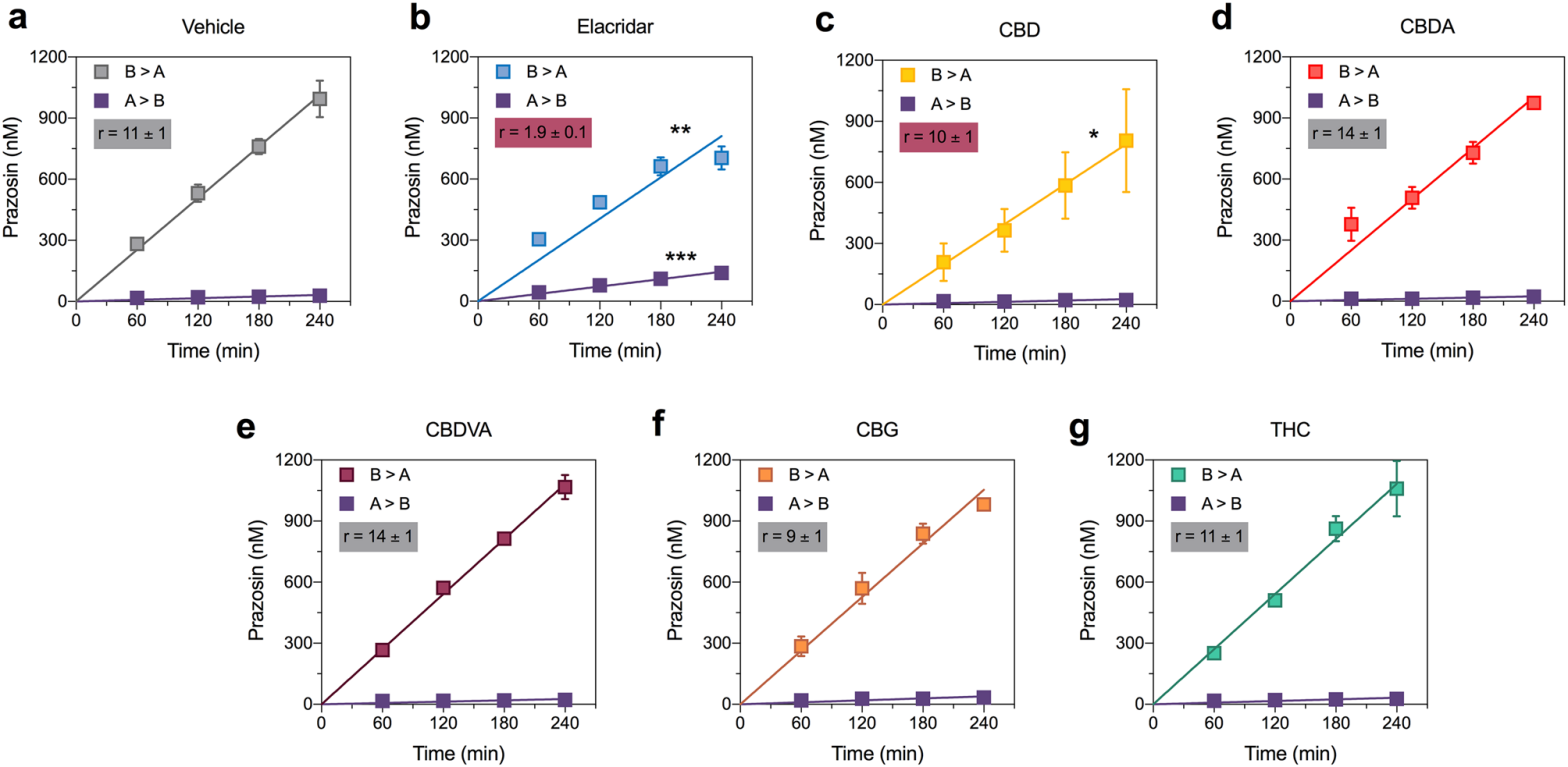
CBD inhibits BCRP-mediated transport. Concentration–time curves for prazosin in the presence of (**a**) vehicle, (**b**) elacridar, (**c**) CBD (**d**) CBDA, (**e**) CBDVA, (**f**) CBG or (**g**) Δ^9^-THC in the basolateral to apical (B > A) and apical to basolateral (A > B) directions in cells expressing BCRP. Elacridar and CBD significantly inhibit (red shading) transport of prazosin. Data are expressed as means ± SEM, with n = 3–4 per time point. Curves represent fits to a linear regression and transport efflux ratios (r) are listed (*p < 0.05, **p < 0.005, ***p < 0.0005 compared to vehicle; Extra sum-of-squares F test).

We also assessed substrate specificity of inhibition by assessing whether the BCRP substrates CBDA, CBG, Δ^9^-THC, CBD and CBDVA (10 µM) similarly inhibited transport of the established BCRP substrate prazosin. Elacridar, the positive control BCRP inhibitor significantly reduced the transport ratio of prazosin compared to vehicle (B > A, *p* = 0.0006; A > B, *p* < 0.0001; Fig. 4a,b; Table 4). Interestingly, CBD was the only cannabinoid to significantly reduce the permeability of prazosin in the B > A direction (*p* = 0.0284) so was identified as an inhibitor of BCRP (Fig. 4, Table 4).

**Table 4:** Permeability of prazosin in the presence of cannabinoids.

	BCRP, prazosin		
Inhibitor	P (B > A)	P (A > B)	r
Vehicle	189 ± 7	18 ± 2	10.5 ± 0.1
CBD	147 ± 20*	15 ± 3	9.9 ± 0.2
CBDA	187 ± 10	13 ± 3	14.0 ± 0.2
CBDVA	201 ± 4	15 ± 3	13.9 ± 0.2
CBG	196 ± 8	22 ± 2	9.1 ± 0.1
Δ^9^-THC	201 ± 8	18 ± 2	11.2 ± 0.1
Elacridar	151 ± 7**	81 ± 4***	1.9 ± 0.1

*P* Permeability calculations (× 10^−2^ cm/s).

*n.d.* not determined; slope of concentration–time curve was not significantly different from zero.

*p < 0.05, **p < 0.005, ***p < 0.0005 compared to corresponding vehicle condition.

**Figure 5:**
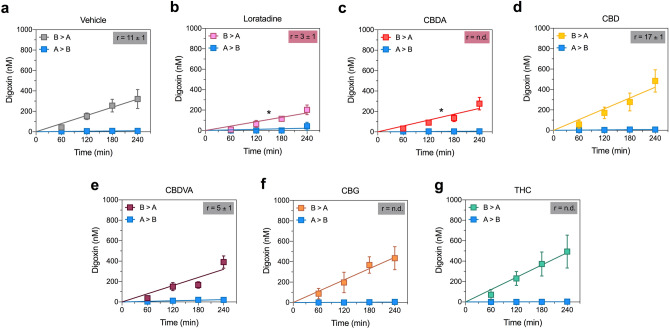
CBDA inhibits P-glycoprotein-mediated transport. Concentration–time curves for prazosin in the presence of (**a**) vehicle, (**b**) loratadine, (**c**) CBDA (**d**) CBD, (**e**) CBDVA, (**f**) CBG or (**g**) Δ^9^-THC in the basolateral to apical (B > A) and apical to basolateral (A > B) directions in cells expressing BCRP. Elacridar and CBD significantly inhibit (red shading) transport of prazosin. Data are expressed as means ± SEM, with n = 3–4 per time point. Curves represent fits to a linear regression and transport efflux ratios (r) are listed (*p < 0.05, compared to vehicle; Extra sum-of-squares F test).

The effect of the five BCRP substrates on P-glycoprotein function was also examined using digoxin as a substrate (Fig. 5, Table 4). Interestingly, CBDA was not a substrate of P-glycoprotein but was an inhibitor as it was the only cannabinoid to significantly reduce the permeability of digoxin in the B > A direction (*p* = 0.0481).

## Discussion

Here we provide evidence for pharmacokinetic interactions between cannabinoids within a full-spectrum cannabis extract. The pharmacokinetic profiles of the cannabinoids when administered in a cannabis extract were markedly different to those when delivered as individual compounds at equivalent doses. Notably, CBDA plasma concentrations were substantially increased, with the total CBDA plasma exposure being 14-fold higher when administered in a cannabis extract than when administered as a single molecule. Conversely, the peak plasma concentrations of the other cannabinoids (e.g. CBD, Δ^9^-THC and Δ^9^-THCA) were considerably lower. The dramatic increase in plasma CBDA exposure likely results, at least in part, from cannabinoid-cannabinoid interactions at the ABC transporter BCRP in the intestinal lumen. We found that CBDA was a substrate of BCRP and that its transport was inhibited by Δ^9^-THC and CBG in vitro. Since CBDA is a BCRP substrate, systemic absorption of orally administered CBDA would be limited by BCRP transporters located in the apical membrane of the intestine. CBG or Δ^9^-THC inhibiting BCRP-mediated CBDA efflux back into the intestinal lumen would result in increased plasma concentrations of CBDA (Fig. 3g).

Our results here provide a potential mechanism explaining the high plasma CBDA concentrations observed following oral dosing of cannabis oils in a human study^5^. This study measured concentrations of CBD and CBDA in biological fluids of healthy individuals treated with an oral cannabis decoction and oil. Serum CBDA concentrations were approximately 20–30 times higher than serum CBD concentrations despite the products containing only 3–6 times the amount of CBDA compared to CBD^5^. A future human pharmacokinetic study could be conducted to test whether our results in mice translate to humans by utilising a similar study design. That is, plasma CBDA concentrations could be compared following oral administration of a CBD dominant cannabis oil versus a purified CBDA oil.

CBD-dominant cannabis-based nutraceutical oils are increasingly being used worldwide with users suggesting that they are effective in treating numerous ailments^11^. Our data suggest that CBDA might have a more significant contribution to the pharmacological effects of these nutraceutical products than previously thought. Given the emerging preclinical evidence that CBDA has anxiolytic, anti-inflammatory, anticonvulsant and antiemetic properties, it is plausible that CBDA might contribute to any medicinal properties of these cannabis-based products^22–25^. However, future placebo-controlled randomized trials are required to examine whether CBDA has therapeutic effects in humans. Artisanal cannabis oils are being used to treat seizures in children with intractable epilepsies. However, as yet, there has been no satisfactory explanation for how these oils exert anticonvulsant effects since the CBD doses administered in these oils are substantially lower than those reported to be effective in reducing seizures in clinical trials^10, 26, 27^. Given that CBDA is anticonvulsant in the *Scn1a*^+/−^ mouse model of Dravet syndrome, the present results suggest CBDA might contribute to the anticonvulsant effects of orally administered artisanal cannabis extracts^24^. Conversely, CBDA might contribute to the adverse effects of these oils, as its safety profile is not well understood.

In vitro transwell assays were used to determine whether ten cannabinoids were substrates of human BCRP and P-glycoprotein. CBD, CBDA, CBDVA, CBG and Δ^9^-THC were identified as substrates of BCRP. The identification of Δ^9^-THC as a BCRP substrate confirms a prior study in mice showing Δ^9^-THC was a Bcrp1 substrate^15^. That study also reported that Δ^9^-THC was a P-glycoprotein substrate (Mdr1a/Mdr1b) in mice, which is inconsistent with the present findings showing Δ^9^-THC is not a human P-glycoprotein substrate^15^. Moreover, CBD was not a substrate of mouse Bcrp1 but was demonstrated to be a human BCRP substrate here^28^. These inconsistencies suggest some caution when comparing mouse and human data on ABC transporter substrates. The identification of CBDA as an ABC transporter substrate is somewhat consistent with previous work where its brain-plasma ratio was significantly increased in a Tween-based vehicle compared to a vegetable oil vehicle^24^. Since non-ionic surfactants such as Tween80 are known to inhibit ABC transporters, inhibition of CBDA efflux by BCRP at the blood–brain barrier by Tween80 is a possible mechanism for the increased brain permeability^29^.

We also determined whether the cannabinoids were inhibitors of BCRP and P-glycoprotein. Previous in vitro and ex vivo studies reported that CBD, Δ^9^-THC and cannabis-based products inhibit BCRP^17–19^. Consistent with these previous studies, CBD inhibited BCRP-mediated transport of prazosin. Surprisingly, CBD did not inhibit BCRP-mediated transport of CBDA; whereas, Δ^9^-THC and CBG inhibited CBDA but not prazosin transport by BCRP. These results further reinforce the importance of considering substrate specificity when evaluating potential transporter-mediated drug-drug interactions (DDIs), as substrate-specific inhibition is a common observation for BCRP and P-glycoprotein^21, 30–33^. Binding sites and affinities of both the substrate and the inhibitor contributed to substrate-dependent interactions, especially with the ABC transporters for which multiple binding sites have been proposed^30, 33–35^. The multiple drug binding sites of P-glycoprotein may explain why CBDA inhibited digoxin transport via P-glycoprotein but was not itself a substrate. Several P-glycoprotein inhibitors with allosteric mechanisms of action such as reduced substrate affinity, decreased ATPase activity, conformational changes that prevent substrate translocation and reduced rates of dissociation have been identified^36–39^. Future studies could explore whether CBDA is a non-competitive inhibitor of P-glycoprotein and its mechanism of transporter inhibition.

Digoxin, a P-glycoprotein substrate with a narrow therapeutic window, has been implicated in several transporter-mediated DDIs. Cardiac and gastrointestinal toxicity has been reported for digoxin when co-administered with P-glycoprotein inhibitors such as quinidine^40–42^. Since DDIs occurring at ABC transporters can have serious clinical consequences, an integral part of the drug development process for new candidates is to evaluate whether they are substrates, inhibitors and/or inducers of ABC transporters as required by drug regulatory agencies^43^. Here, only four of the cannabinoids (CBD, CBDA, CBG and Δ^9^-THC) inhibited ABC transporter function, suggesting the likelihood of transporter-mediated DDIs by cannabinoids is low. However, before a definitive conclusion on DDI liability can be made, additional distinct probe substrates should be screened so any potential DDIs resulting from substrate specificity are not overlooked.

While DDIs occurring at ABC transporters can have serious adverse effects, inhibition of transporters can also be therapeutically advantageous. Many anticancer and antimicrobial drugs are substrates of ABC transporters and, therefore, have low bioavailability^44–46^. Rational drug design efforts have involved non-toxic BCRP and P-glycoprotein inhibitors, including excipients, to purposefully enhance oral absorption of substrates^36, 46–50^. A similar therapeutic advantage of cannabinoids improving the low bioavailability of co-administered therapeutic drugs that are ABC transporter substrates could be explored in future studies.

Several limitations of the present study need to be considered. While not examined here, terpenoids and flavonoids inhibit ABC transporters and could contribute to the increased absorption of CBDA within the full-spectrum extract^51, 52^. Moreover, interactions mediated by the cytochrome P450 (CYP450) family of drug metabolizing enzymes could also contribute to pharmacokinetic entourage effects in cannabis. CYP450s tend to be the most common source of pharmacokinetic interactions since competition between two drugs for the same metabolizing pathway can drastically affect metabolism and elimination parameters. While the metabolic pathways are still unknown for many of the cannabinoids, CYP450-mediated metabolism contributes extensively to the elimination of CBD and Δ^9^-THC^53^. The prolonged t_1/2_ values observed for the cannabinoids when administered as a full-spectrum extract could be the consequence of interactions between the cannabinoids at the drug metabolizing CYP450 enzymes. Indeed, we recently reported that several phytocannabinoids found in the full-spectrum extract inhibited CYP450 enzymes including CYP3A4, CYP2C9, CYP1A2, CYP2B6 and CYP2C19^54^. While the CYP450 enzymes involved in the metabolism of CBDA have not been characterized, it is possible that cannabinoid inhibition of CYP450-mediated first-pass metabolism of CBDA could account for its increased plasma concentrations when administered as a full-spectrum extract compared to as an individual cannabinoid. In any case, the present observation of cannabinoid-CBDA interactions at the ABC transporter BCRP are very likely to contribute to the enhanced plasma CBDA concentrations that were observed following oral administration of a cannabis extract.

## Conclusion

Many have been puzzled by the high bioavailability of CBDA in humans following the oral ingestion of CBD-dominant cannabis-derived nutraceutical oils^5, 55–57^. Our results suggest that the oral administration of such cannabis extracts provides a natural vehicle to enhance plasma CBDA concentrations due to cannabinoid-cannabinoid interactions at the drug efflux transporter BCRP. Taken together with emerging preclinical evidence that CBDA has anti-emetic, anxiolytic and anticonvulsant effects, the present results highlight that the contribution of CBDA to the pharmacological effects of hemp nutraceutical products warrants further inspection. Our results showing pharmacokinetic interactions between cannabinoids provides one mechanism for the much touted "entourage effect" of cannabis.

## Methods

### Drugs

CBD, CBDA, CBDV, CBG, CBGA, Δ^9^-THC and Δ^9^-THCA were purchased from THC Pharm GmbH (Frankfurt, GER). CBGA was also purchased from Cayman Chemical (Ann Arbor, USA). CBDA was also generously provided by Medropharm GmbH (Schönenberg, CHE). CBDVA was synthesized by Professor Michael Kassiou at the University of Sydney. Elacridar, digoxin and loratadine were purchased from MedChem Express LLC (Princeton, USA). Prazosin and Lucifer Yellow CH dipotassium salt were purchased from Sigma-Aldrich (St. Louis, USA). The full-spectrum cannabis extract was purchased from Ecofibre (Brisbane, AUS) with its cannabinoid content presented in Supplemental Table 1. Terpene content of the full-spectrum extract in percent content (w/w) is as follows: β-caryophyllene, 0.525; β-linalool, 0.011; D-limonene, 0.006 and β-pinene, 0.004.

### Animals

All animal care and procedures were approved by the University of Sydney Animal Ethics Committee (protocols 2016/1036 and 2017/1292) in accordance with the Australian Code of Practice for the Care and Use of Animals for Scientific Purposes in compliance of the ARRIVE guidelines. Experimental mice were generated by breeding 129S6/SvEvTac (Australian BioResources; Moss Vale, AUS) with C57BL/6J (Jackson Laboratory stock 000664; Animal Resources Centre; Canning Vale, AUS). Mice were group-housed under a 12 h light/12 h dark cycle with ad libitum access to food and water.

### Pharmacokinetic study

The full-spectrum cannabis extract and individual cannabinoids (CBD, CBDA, CBDVA, CBGA, Δ^9^-THC, Δ^9^-THCA) were prepared fresh on the day of the experiment as solutions in hemp seed oil (Hemp Foods Australia Pty Ltd; Bungalow, AUS). Hemp seed oil was devoid of any cannabinoids. Drugs were administered by oral gavage in a volume of 5 mL/kg. Wildtype male and female mice (postnatal day 21–28, P21-28) received a single oral gavage of either the full-spectrum cannabis extract or an individual cannabinoid. Cannabinoid doses within the full-spectrum extract or as an individual cannabinoid were as follows in mg/kg: CBDA and Δ^9^-THCA, 50; CBD, 25; Δ^9^-THC, 15; CBC, 9; CBDVA and CBGA, 7; CBN, 2.5; CBG, 1.5; CBDV and Δ^9^-THCV, < 1 (Fig. 1a). At selected time points (15–240 min), mice were anesthetized with isoflurane and whole blood was collected by cardiac puncture. Plasma was isolated by centrifugation (9000 g for 10 min, 4 °C) and stored at − 80 °C until assayed.

### Analytical methods

Concentrations of cannabinoids in plasma samples were quantified as described previously^8, 24, 58^. Briefly, plasma samples were spiked with diazepam as an internal standard and protein precipitation was achieved by vortex-mixing with acetonitrile. The organic layer was isolated by centrifugation (4000* g* for 10 min) and evaporated to dryness with N_2_. Samples were reconstituted in acetonitrile and 0.1% formic acid in water (1:3.3, v/v) for supported-liquid extraction (SLE) with methyl *tert*-butyl ether (MTBE) using Biotage Isolute SLE+ columns (Uppsala, SWE). Samples were evaporated to dryness with N_2_ and reconstituted in acetonitrile and 0.1% formic acid in water (1:1, v/v) for analysis by LC–MS/MS as previously described^8, 10, 24, 58^. Quantification of cannabinoids was achieved by comparing samples to standards prepared with known amounts of drug.

### Pharmacokinetic calculations

Plasma concentrations at each time point were averaged and pharmacokinetic parameters were calculated by noncompartmental analysis. Elimination rate constants were determined by linear regression of the terminal component of the concentration–time curve using GraphPad Prism 8.2 (La Jolla, USA). The log-linear trapezoidal method was used to calculated total drug exposure (area under concentration–time curve) using equations described previously^59^.

### Cell culture

Madin-Darby Canine Kidney II (MDCK) cell lines were generously provided by Dr. Alfred Schinkel (Netherlands Cancer Institute, NLD) by way of Associate Professor Joseph Nicolazzo (Monash University, AUS). Cell lines included wildtype MDCK cells and MDCK cells stably expressing human MDR1 (P-glycoprotein) or ABCG2 (BCRP). Cells were grown in High-glucose Dulbecco’s modified Eagle’s medium (DMEM) supplemented with 10% fetal bovine serum (FBS), 100 U/mL penicillin and 100 µg/mL streptomycin (P/S) in a humidified 5% CO_2_ atmosphere at 37 °C.

### Bidirectional transport assays

Corning Transwell polycarbonate membrane cell culture inserts (0.4 µm, 6.5 and 12 mm; Corning Inc.; Corning, USA) were used for bidirectional transport assays. Briefly, 72 h prior to the transwell assay, cells (2.5 × 10^5^ cells/well or 2.0 × 10^5^ cells/well for 12 mm or 6.5 mm inserts, respectively) were plated.

For substrate assays, cells were rinsed with PBS and vehicle (DMSO) or 10 µM inhibitor (loratadine, P-glycoprotein or elacridar, BCRP) in DMEM supplemented with 10% FBS was added to both the apical and basolateral chambers and incubated for 15 min at 37 °C in a humidified 5% CO_2_ atmosphere. Media in the donor chamber was then replaced with that containing 10 µM of an individual cannabinoid in the presence of either vehicle or inhibitor and returned to 37 °C. Aliquots (25 or 50 µL) were removed from the accepter chamber at 60, 120, 180 and 240 min.

For inhibitor assays, cells were rinsed with PBS and 10 µM of an individual cannabinoid in DMEM supplemented with 10% FBS was added to both the apical and basolateral chambers and incubated for 15 min at 37 °C in a humidified 5% CO_2_ atmosphere. Following the incubation, media in the donor chamber was replaced with 1 µM substrate (digoxin, P-glycoprotein or prazosin, BCRP) and its respective cannabinoid, returned to 37 °C and aliquots were removed from acceptor chamber as described above.

Concentrations of cannabinoids, digoxin or prazosin in the acceptor chamber were quantified using LC–MS/MS. Samples were spiked with diazepam as an internal standard and then either 0.1% formic acid in water (cannabinoids and digoxin) or 0.5 M sodium hydroxide (prazosin) was added for SLE with MTBE (cannabinoid and digoxin) or ethyl acetate (prazosin). Samples were evaporated to dryness with N_2_ and reconstituted in 1:1, v/v acetonitrile and 0.1% formic acid in water (cannabinoids), methanol and 0.1% formic acid in water (prazosin) or methanol and 0.1% formic acid in 10 mM ammonium acetate (digoxin) for analysis by LC–MS/MS. Cannabinoids were analyzed as described above. The mass spectrometer was operated in positive electrospray ionization mode with multiple reaction monitoring (digoxin: 798.35 > 651.45, 798.35 > 97.15; prazosin: 384.05 > 95.05, 384.05 > 247.1).

### Lucifer yellow permeability assay

At the completion of the transwell assay, a Lucifer yellow permeability assay was conducted to confirm monolayer integrity. High Potassium Hank's Balanced Salt Solution (HBSS) replaced the media in both chambers. Lucifer yellow (250 µM) was added to the apical chamber and cells were incubated at 37 °C for 60 min. A CLARIOstar plate reader (BMG Labtech; Offenburg, GER) was used to take fluorescence readings over 0.5 ms (excitation 485 nm, emission 535 nm) from samples taken from the basolateral chamber. Baseline fluorescence as measured from samples containing HBSS only was subtracted and fluorescence readings were normalized to those of 250 µM Lucifer yellow. Monolayers were considered intact if Lucifer yellow permeability was less than 5%^60^.

### Data analysis

Rates of substrate transport were determined by linear regression of concentration–time curves using GraphPad Prism. Apparent permeability (P) of substrate transport across MDCK cell monolayers were calculated for both the basolateral to apical (B > A) and apical to basolateral (A > B) directions as previously described using the following equation:

$${\text{P}} = \frac{{\text{V}}}{{{\text{C}}_{0} \times {\text{SA}} }} \times \frac{{\Delta {\text{C}}}}{{\Delta {\text{t}}}}$$

V, volume of acceptor chamber (B > A: 0.5 mL and 0.2 mL and A > B: 1.5 mL and 0.6 mL for 12 mm and 6 mm inserts, respectively). C_0_, initial substrate concentration in the donor chamber (10 µM cannabinoids or 1 µM digoxin and prazosin). SA, monolayer growth surface area (1.12 cm^2^, 12 mm inserts; 0.33 cm^2^, 6.5 mm inserts)^21^. ΔC/Δt, slope calculated concentration–time curves. Transport efflux ratios (r) were calculated by dividing the apparent permeability calculated for the B > A direction by that calculated for the A > B direction. Transport ratios could not be calculated in instances when the slope for concentration–time curves in the A > B direction were not significantly different from zero.

Comparisons of curve fits for concentration–time curves between wildtype MDCK cells and MDCK cells expressing P-glycoprotein or BCRP were calculated using the Extra sum-of-squares F test to determine whether a cannabinoid was a substrate. Rates of substrate transport in the A > B direction were not different between wildtype and transporter-expressing cells for any cannabinoid, with the exception of CBG and Δ^9^-THC in cells expressing BCRP. Comparisons of curve fits in the B > A direction with *p* < 0.05 were considered significantly different and indicative of a substrate for the corresponding transporter. In order to determine whether cannabinoids were inhibitors, comparisons of curve fits for concentration–time curves between vehicle-treated and cannabinoid-treated cells were calculated using the Extra sum-of-squares F test. Comparisons of curve fits with *p* < 0.05 were considered significantly different and indicative the compound being an inhibitor.

## Supplementary Information

Supplementary Table.

## Data Availability

All relevant data are presented within the manuscript and are available from the corresponding author on reasonable request.
